# Investigating the effects of low intensity visible light on human keratinocytes using a customized LED exposure system

**DOI:** 10.1038/s41598-022-23751-3

**Published:** 2022-11-07

**Authors:** Emily Sutterby, Chanly Chheang, Peter Thurgood, Khashayar Khoshmanesh, Sara Baratchi, Elena Pirogova

**Affiliations:** 1 School of Engineering, RMIT University, Melbourne, VIC Australia; 2 School of Health and Biomedical Sciences, RMIT University, Bundoora, VIC Australia

**Keywords:** Biomedical engineering, Cell biology, Lasers, LEDs and light sources

## Abstract

Photobiomodulation (PBM) refers to the use of light to modulate cellular processes, and has demonstrated utility in improving wound healing outcomes, and reducing pain and inflammation. Despite the potential benefits of PBM, the precise molecular mechanisms through which it influences cell behavior are not yet well understood. Inconsistent reporting of key light parameters has created uncertainty around optimal exposure profiles. In addition, very low intensities of light, < 0.1 J/cm^2^, have not been thoroughly examined for their use in PBM. Here, we present a custom-made compact, and modular LED-based exposure system for studying the effects of very low-intensity visible light (cell proliferation, migration, ROS production, and mitochondrial membrane potential) of three different wavelengths in a parallel manner. The device allows for six repeats of three different exposure conditions plus a non-irradiated control on a single 24-well plate. The immortalised human keratinocyte cell line, HaCaT, was selected as a major cellular component of the skin epidermal barrier. Furthermore, an in vitro wound model was developed by allowing the HaCaT to form a confluent monolayer, then scratching the cells with a pipette tip to form a wound. Cells were exposed to yellow (585 nm, 0.09 mW, ~ 3.7 mJ/cm^2^), orange (610 nm, 0.8 mW, ~ 31 mJ/cm^2^), and red (660 nm, 0.8 mW, ~ 31 mJ/cm^2^) light for 10 min. 48 h post-irradiation, immunohistochemistry was performed to evaluate cell viability, proliferation, ROS production, and mitochondrial membrane potential. The results demonstrate increased proliferation and decreased scratch area for all exposure conditions, however only red light increased the mitochondrial activity. Oxidative stress levels did not increase for any of the exposures. The present exposure system provides opportunities to better understand the complex cellular mechanisms driven by the irradiation of skin cells with visible light.

## Introduction

Human skin is a complex, multilayered organ that interacts with a plethora of external stimuli^1,2^. Skin is our first line of defence, offering non-specific protection against a plethora of pathogens and potentially harmful chemicals. However, when the skin barrier is breached by cuts and wounds, the body is left vulnerable to infection. The skin has some regenerative abilities, recruiting multiple cell types from different layers to restore barrier function^2^. However, when wounds exceed approximately 1 mm deep, the healing process becomes difficult and scar tissue tends to form^3^. Furthermore, infected non-healing wounds present a significant burden on both the individual and the healthcare economy, costing an estimated $50 billion in the United States alone^4,5^. Existing treatment options have limited efficacy, leaving a gap for more effective therapies to fill.

Non-ionising electromagnetic radiation (EMR) in the visible light spectrum has been used as a therapeutic modality to improve wound and soft tissue healing, reduce the appearance of aging, increase the rate of hair growth, reduce pain and inflammation, and has potential as an alternative cancer therapy^6–11^. More recently, light has demonstrated utility during in vitro cell and tissue culture, acting as an antioxidant substitute post-thawing of human stem cells and accelerating the formation of a dermal equivalent for human skin modelling^12,13^. Previously referred to as low-level laser therapy (LLLT), the use of light in biology is now more accurately referred to as photobiomodulation (PBM). This definition aims to encompass light from different sources, including light emitting diodes (LEDs), and to acknowledge the potential inhibitory or non-therapeutic effects of light stimulation^14^.

The wavelength of EMR plays a key role in determining the PBM effect. Violet and blue light (380–500 nm), which have a shorter wavelength and contain more energy than red light, have demonstrated antibacterial properties without slowing wound healing^15^. Meanwhile red (620–750 nm) and near infrared (750–950 nm) light have been used as a therapeutic modality to reduce signs of photoaging and encourage wound healing^16^. A promising modality for treating infected, non-healing wounds is to first reduce the bacterial load by exposure to blue light, followed by exposure to red light to promote wound closure^15,17^.

Red and near-infrared light are commonly posited to interact with chromophore cytochrome c oxidase (CCO)^18–20^. CCO is the terminal enzyme of the respiratory chain, and its stimulation leads to a reported increase in ATP synthesis^21,22^. While many researchers corroborate the involvement of CCO in PBM, a recent study found that cell lines lacking CCO still exhibited increased proliferation during PBM, thus suggesting that multiple pathways are likely targeted^23^. Meanwhile, blue and violet light are posited to act on light-gated ion channels within cells, and higher wavelengths in the infrared region are posited to target water as a chromophore promoting proliferation via the TRPV1 calcium ion channel pathway^24,25^.

While the effects of visible light on cell metabolism were demonstrated before, the fundamental mechanisms of action through which light irradiation acts are not comprehensively understood^26^. It appears multiple pathways may be activated, with the induced effects being highly dependent on the wavelength and intensity of light applied^27^. It is likely that different wavelengths of light interact with different chromophores and thus, affect different cellular pathways. Dose of irradiation also plays a central role in determining PBM outcomes. In addition to the energy density and duration of exposure, dose is dependent on the absorption of energy by the target biological media. The optimal energy density appears to be in the range of 0.1–5.0 J/cm^2^, with densities above 10–15 J/cm^2^ considered less effective or resulting in cell death^28,29^. Densities below 0.1 J/cm^2^ have not been thoroughly investigated, which is of particular interest to this study.

Previously, it was believed that only lasers could provide sufficient energy to stimulate cells, with almost all research on PBM in the 80-90 s conducted using lasers^14,30^. Lasers produce a focused light source in both coverage and spectral bandwidth, emitting monochromatic, coherent light^14^. While this precise control over the emission frequency may be beneficial for certain applications, for PBM therapy it appears that a range of frequencies have beneficial effects, and as such, having tight control over the spectral bandwidth may not be necessary for practical applications^24^. Conversely, LEDs have a broader spectral bandwidth and can emit light over a larger surface area. This property can be beneficial, when covering large areas of tissue, as multiple LEDs (LED array) can be configured to increase the total beam area^14^. In addition, LEDs are typically inexpensive compared to lasers, more efficient and can be incorporated into wearable or handheld lightweight devices for home-use applications^31^.

In vitro PBM studies often rely on the use of commercially available LED lamps, which can be expensive, rely on mains power supply and often do not allow for different wavelengths to be generated and studied simultaneously^32,33^. Hand-held devices further suffer from inconsistent placement in relation to the sample, subsequently altering the delivered dose. Custom-built LED arrays, or LED tables, circumvent some of these limitations. However, they are often rigid in design, allowing limited control of the specific wavelength, pulse rate, intensity of light, and are reliant on mains power supply. Illumination often comes from underneath the well plate, making them potentially unsuitable for more complex full-thickness skin models, where top-down illumination may be important to mimic real-world (in vivo) conditions, particularly for blue light as it has a shallow penetration depth^12,34,35^. Further, inconsistent reporting of key light exposure parameters, including dosimetry and experimental conditions, limit the repeatability of experiments and introduce uncertainties when comparing the literature^28^. Perhaps owing to these inconsistencies, optimal exposure parameters, including wavelength, irradiance, fluence, pulse rate, and dose are not widely agreed upon^8^.

Herein, we demonstrate a customizable, low-cost and compact exposure system for studying the impact of low-intensity visible light on human keratinocytes. The system is compatible with standard 24-well cell culture plates and is amenable for parallel experiments. The system, comprised of an array of LEDs, is battery powered and can be modulated for continuous or pulsed exposure with adjustable intensities. Furthermore, the LEDs may be swapped out, making it possible for different wavelengths of light to be implemented. The developed device is suspended on top of a standard 24-well plate rather than applying light from the bottom of the plate. Proof-of-concept experiments have been conducted to study the proliferation, wound healing rate and mitochondrial membrane potential in the immortalized human keratinocyte cell line HaCaT. Very low doses of light irradiation were applied (energy density < 0.032 J/cm^2^ and exposure duration of 600 s) to investigate whether these low doses contain sufficient energy to stimulate HaCaT cells. Red light is most commonly used for PBM; however, the effects of orange and yellow light are less well understood. Thus, in addition to the red light (660 nm), shorter wavelengths of 610 nm (orange), and 585 nm (yellow), were selected to investigate the influence of wavelengths on PBM outcomes. The present results demonstrate the suitability of the customised LED system for in vitro evaluation of different light exposures on selected cells, thus providing opportunities to better understand the complex cellular mechanisms driven by the irradiation of skin cells with visible light.

## Principles of the exposure system

**Figure 1:**
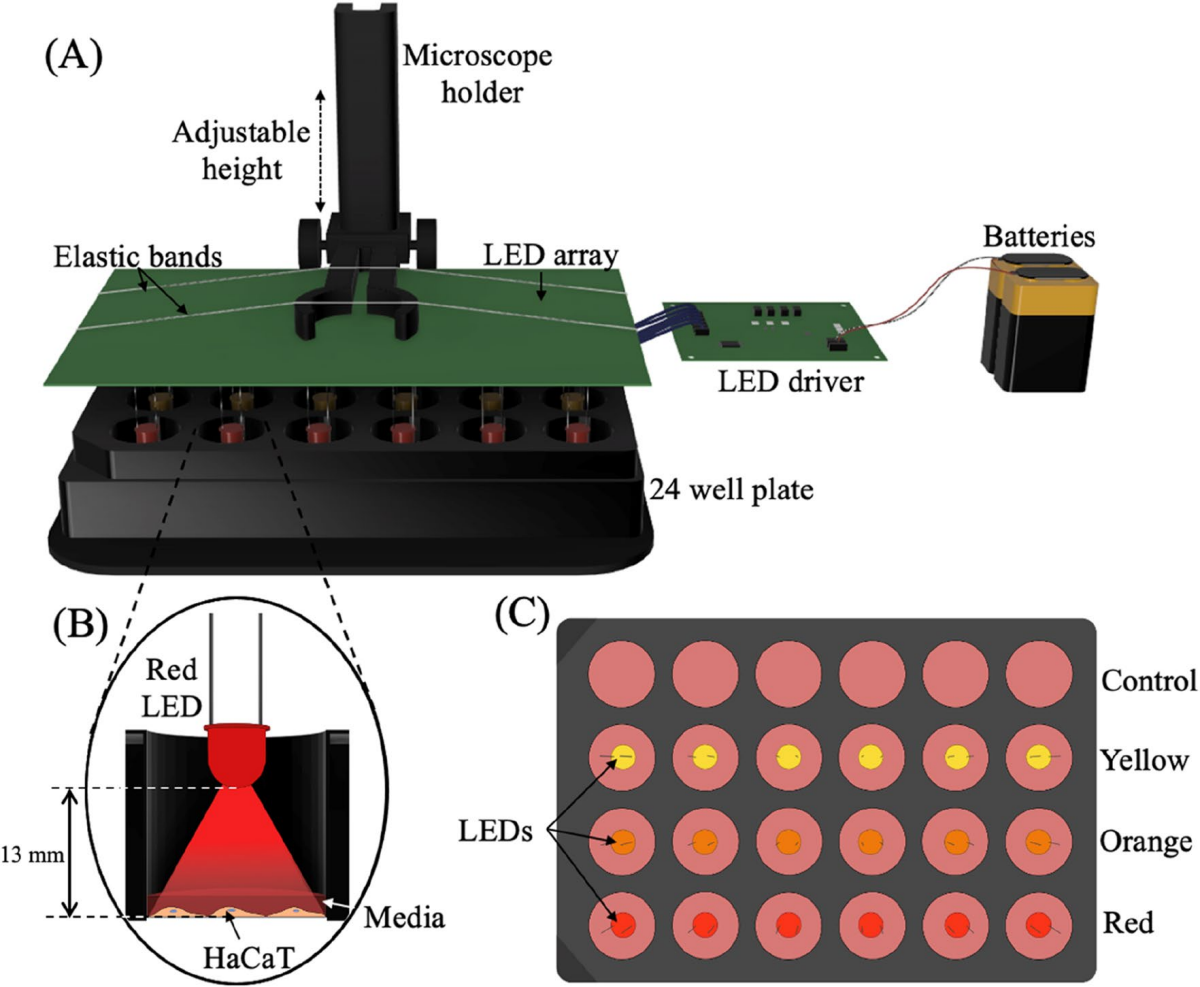
Visible light exposure system. (**A**) Schematic showing the experimental set up, comprised of an LED array, LED driver, two 9 V and one 1.5 V batteries, and a microscope holder to position the LEDs above an opaque 24-well plate. (**B**) Cross-section of the inside of a well demonstrating position of LED and visible light exposure. There is a 13 mm gap between the LED tip and the HaCaT monolayer. (**C**) Top-down view of the LEDs and 24-well plate. Multiple different wavelength LEDs and sham exposures can be run in parallel on a single plate. The configuration for the experiments described herein include a control/sham condition, yellow light (585 nm), orange light (610 nm), and red light (660 nm).

The exposure system is comprised of an LED array suspended above a 24-well cell culture plate, an LED driver, and two 9 V and one 1.5 V batteries (Fig. 1A). The array consists of 18 through-hole LEDs (3 mm, Lumex QuasarBrite™) spaced to align centred within the wells of a standard 24-well cell culture plate at a heigh of 13 mm from the tip of the LED to the HaCaT monolayer, as shown in Fig. 1B. A three-channel LED driver (MAX16823, Maxim) ensures a stable current supply to the LEDs from a battery power supply.

The driver allows the current of each channel to be modulated independently of each other via three current sensing resistors. Each channel of driver may also be supplied with a pulse width modulated signal to pulse the LEDs. Furthermore, the individual LEDs can be swapped out to facilitate different wavelengths of light including UV and IR, although the battery supply may need to be adjusted to meet the forward voltage for different types of LED. The separate channels supply power to 6 LEDs each, and the pulse rate and intensity of each channel can be tuned separately, thus facilitating multiple exposure conditions to be generated concurrently in separate wells of a single 24-well plate. Hence, the system enables three different exposure conditions plus a control with 6 repeats to be investigated on a single 24-well cell culture plate as demonstrated in Fig. 1C. The overall cost of the system is ~ $90 AUD, as detailed in the supplementary information S1.

The system is set up first by sterilizing the LED array with ethanol, then placing the array inside a laminar flow hood and UV sterilizing for 15 min. Then, top-down exposure is achieved by suspending the LED array above the 24-well plate using a microscope holder, which enables the height of the LEDs within the well to be adjusted. A major shortcoming of handheld devices is inconsistent spacing between cells and the exposure device, a limitation circumvented via the proposed system. This will also be advantageous for complex, 3D-tissue models where the top-down orientation of exposure is important in mimicking in vivo conditions. Finally, the lid of the 24 well plate containing the cells is placed underneath the array, and the array is lowered to a height of 13 mm above the cells and the battery is connected to the driver to turn on the LEDs.

The viewing angle of the LEDs was optimised to ensure even coverage of at the bottom of the well. Opaque 24-well plates (Black VisiPlate, PerkinElmer) are used to ensure no crosstalk between the LEDs (no overlap of light exposure between the wells). The compact size of the system enables it to be placed inside a cell incubator during longer exposure durations, though the batteries should remain outside or be hermetically sealed due to the humidity.

The inexpensive and modular exposure system enables customizable exposure conditions to be generated, providing the user with control over the distance between the light source and cells (density of exposure), and control over the intensity, pulse rate and wavelength of the light emitted. Furthermore, compatibility with a 24-well plate enables repeats of each condition to be conducted on a single plate, hence increasing throughput and streamlining experiments.

## Results

### Visible light exposure profile

**Figure 2:**
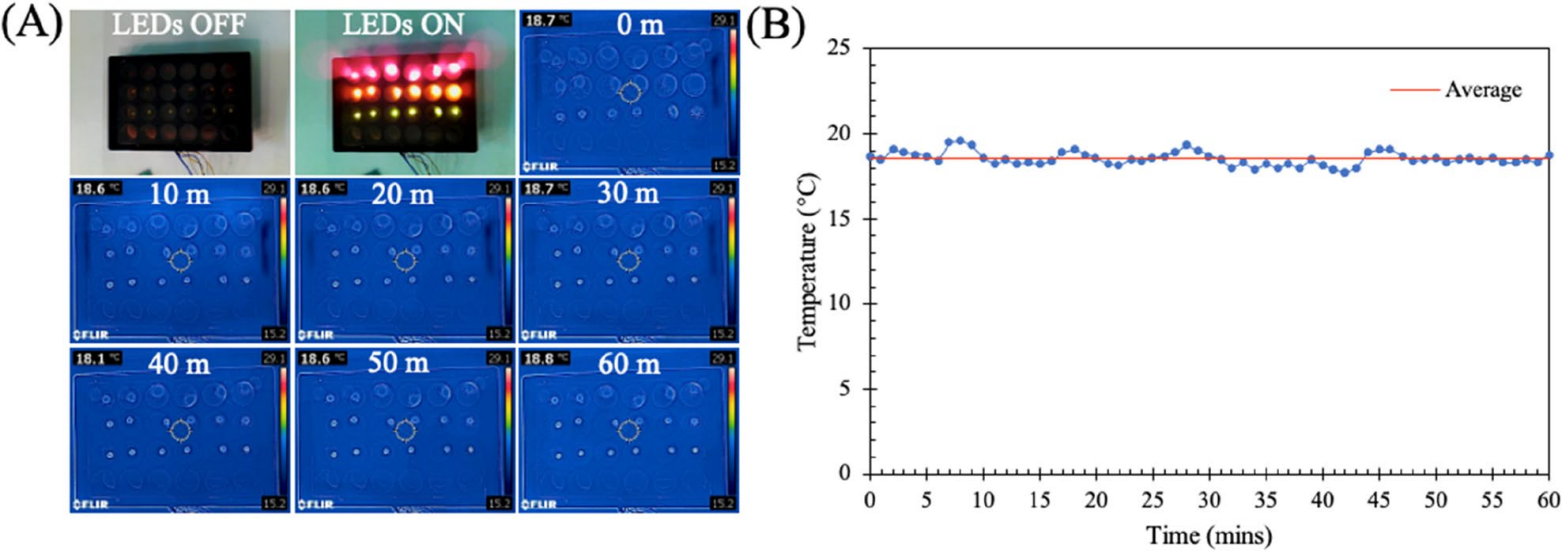
Characterisation of temperature change from LEDs using a thermal camera. Images taken every minute for 60 min. (**A**) Thermal images of LED array in 24-well plate from time 0 min to 60 min. (**B**) Graph showing temperature variation as measured by the thermal camera.

It was critical to ensure that any effects observed were caused exclusively by the electromagnetic radiation emitted by the LEDs and not heating effects. Thus, a thermal camera was used to observe the temperature profile over time (Fig. 2A). Images were captured every minute over a period of 60 min and the results, quantified and shown in Fig. 2B, indicate an average temperature of 18.54 ± 0.4 °C. Importantly, for the exposure conditions used there is minimal variation in temperature, indicating a non-thermal mechanism of action. Furthermore, the absorption of light by the PBS used to cover the cells during exposure, and of the cells themselves was measured as described in S4. The results demonstrate that very little light is absorbed by either the PBS (< 8%) or the cell monolayer (< 6%).

### Irradiation increases proliferation in human keratinocytes

Initially, we assessed the effect of low-intensity visible light on the viability of HaCaT cells using a dead/live assay with DAPI staining all cells and PI staining non-viable cells. The percentage of viable cells could then be calculated by dividing the total number of cells minus the non-viable cells by the total number of cells. The energy density was set to 3.70, 31.12, or 31.36 mJ/cm^2^ while the wavelength was set to 585, 610, and 660 nm, respectively. A control group (non-irradiated cells) was exposed to the same experimental conditions as the irradiated experimental groups. A positive control (non-viable HaCaT exposed to ethanol) was employed to ensure the PI stain was binding to dead cells. 48 h after a single 10-min exposure, no significant changes in cell viability were observed compared to the control group (Supplementary Figure S2).

Next, we evaluated the effect of low-intensity visible light on the proliferation of HaCaT cells 48 h following exposure using a BrdU assay. The same exposure conditions as used for the viability assay were used. BrdU is a thymidine analogue that incorporates into proliferating cells during DNA synthesis^36^. The intensity of the developed BrdU is directly proportional to the quantity of BrdU in the cells, thus a greater intensity indicates increased proliferation. The 48 h between exposure and fixing provided sufficient time for BrdU to integrate into the cells.

**Figure 3:**
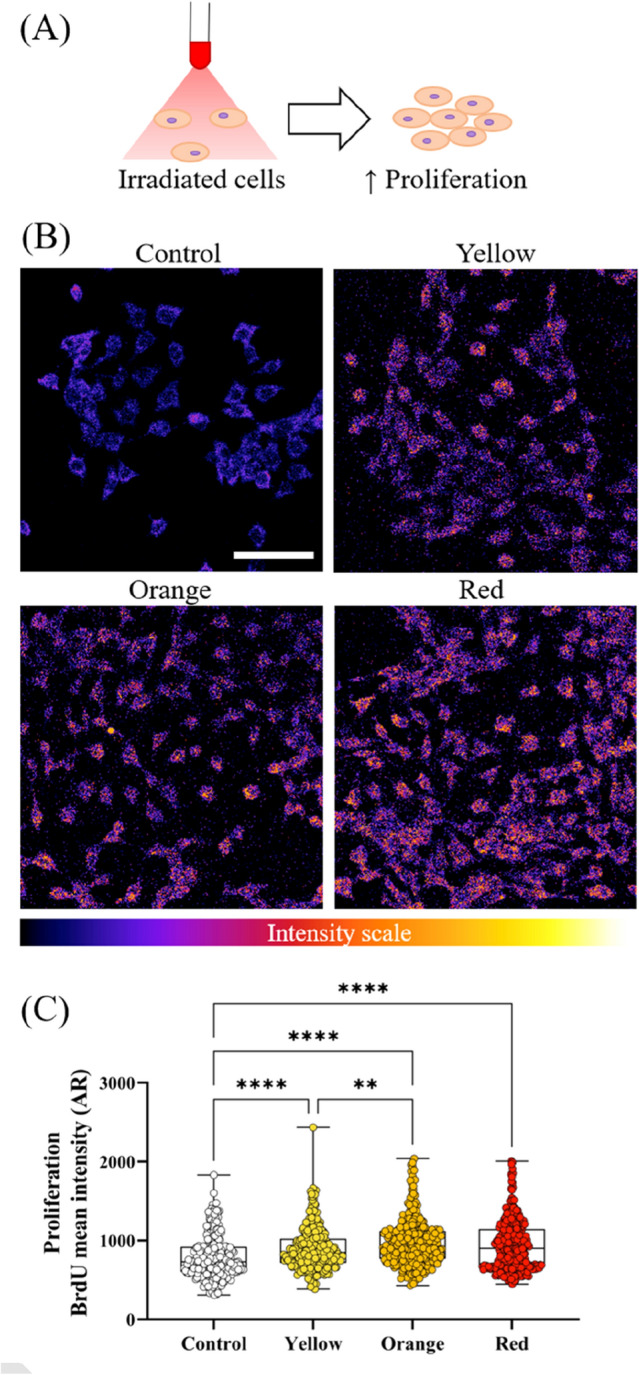
Effect of visible light exposure on HaCaT proliferation. (**A**) Immunofluorescent images of BrdU incorporation into HaCaT cells. BrdU intensity is shown as a colour scale with low intensities appearing more blue and high intensities appearing more purple and orange. (**B**) HaCaT proliferation increases when exposed to visible light. (**C**) Quantitative analysis of BrdU intensity. Each dot represents a single cell and at least 240 cells across four independent experiments have been analysed. Boxes show median, first and third quartiles. Scale bar in B represents 100 μm. **P < 0.01, ****P < 0.0001, ordinary one-way ANOVA multiple comparisons test.

Immunofluorescent images of BrdU intensity (Fig. 3B) were quantified and displayed in Fig. 3C. Using this approach, we found that all three exposure conditions significantly improved the density of BrdU positive cells, with a fold increase of 1.14 ± 0.34 for yellow, 1.24 ± 0.38 for orange and 1.19 ± 0.40 for red compared to the control. These results indicate that even at very low doses, visible light is capable of increasing HaCaT proliferation (Fig. 3A).

### Wound healing time reduced in HaCaT cells exposed to visible light

To assess the impact of low intensity light on HaCaT wound healing in vitro, a 2D scratch assay was performed. A single 10-min LED exposure with an energy density of 31.36 mJ/cm^2^ at 660 nm, 31.12 mJ/cm^2^ at 610 nm, or 3.70 mJ/cm^2^ at 585 nm was imparted on cells cultured in a black 24-well plate immediately after scratching with a 1000 μl pipette tip.

**Figure 4:**
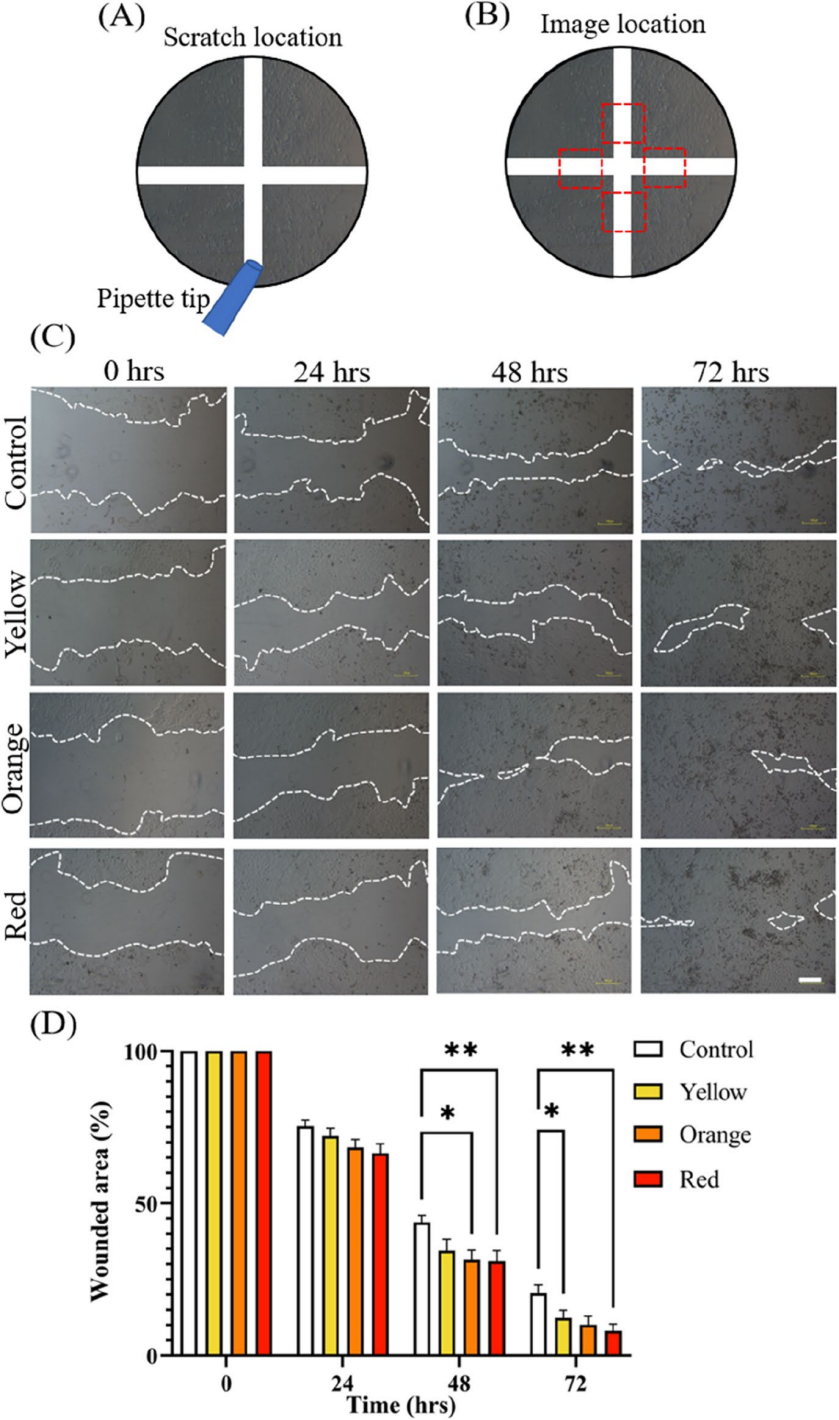
Measurement of HaCaT wound healing when exposed to visible light in in vitro scratch assay. (**A**) Schematic demonstrating location of the scratches made using a 1000 μl pipette tip. (**B**) Schematic highlighting location of images to both sides of the vertical scratch as indicated by the red boxes. (**C**) Representative images of wounded HaCaT monolayers for each visible light condition (red, orange, yellow) and control. Images captured at 0, 24, 48 and 72 h. Dotted lines define regions lacking cells. (**D**) Significant results observed between red light and control conditions at 48 and 72 h time points. Time 0 h is normalised to 1 and further time points are reflected as a portion of time 0 h. Images in 4c are representative images selected from two independent experiments. Scale bar in (**C**) represents 100 μm, error bars in D represent standard error, *P < 0.05, **P < 0.01, two-way ANOVA Tukey’s multiple comparisons test.

A horizontal and vertical scratch was made at the centre of each well as shown in Fig. 4A, and images were taken slightly off centre on each of the scratch lines as highlighted by the red boxes in Fig. 4B. Brightfield microscopy images were taken immediately after the first exposure (0 h) and at 24 h intervals for 72 h as shown in Fig. 4C.

The cell-free area at each timepoint was found using ImageJ and the results shown in Fig. 4D. Exposure to all light conditions increased the rate of wound closure compared to a non-irradiated control, with red and orange light producing the most effective results. Within 48 h of exposure, cells exposed to red light had reduced the size of the wounded area by approximately 69 ± 16% (P < 0.01), orange light by 69 ± 14% (P < 0.05), and yellow by 66 ± 17% (not significant) while the control had achieved a 57 ± 10% reduction. This reduction in wound healing time is indicative of the potential therapeutic benefits of red light on skin wounds.

### Mitochondrial membrane potential increases with exposure to low-intensity red light

Mitochondria contain chromophores (molecules that can absorb and respond to visible light) that are believed to be the primary site of light absorption by cells during red light PBM therapy^22^. Specifically CCO, an enzyme in the mitochondrial respiratory chain, has been shown to be activated by PBM, inhibiting the dissociation of nitric oxide from CCO^20,26,37^. This subsequentially leads to electron transport restoration and increased mitochondrial membrane potential^22^. However, orange and yellow light may act on a different pathway. Thus, we evaluated the impact of yellow light (585 nm, ~ 3.7 mJ/cm^2^), orange light (610 nm, ~ 31 mJ/cm^2^) and red light (660 nm, ~ 31 mJ/cm^2^) on mitochondrial membrane potential. To achieve this, cells were stained with MitoTracker orange prior to exposure, then imaged 48 h later. MitoTracker orange probes bond with mitochondrial thiols in live cells and accumulates with increased mitochondrial membrane potential^38^. Furthermore, ROS is produced as a by-product during activation of the electron transport chain and plays multiple roles in both cellular function and dysfunction^39^. Thus, a ROS assay was conducted in addition to the mitochondrial membrane potential assay.

**Figure 5:**
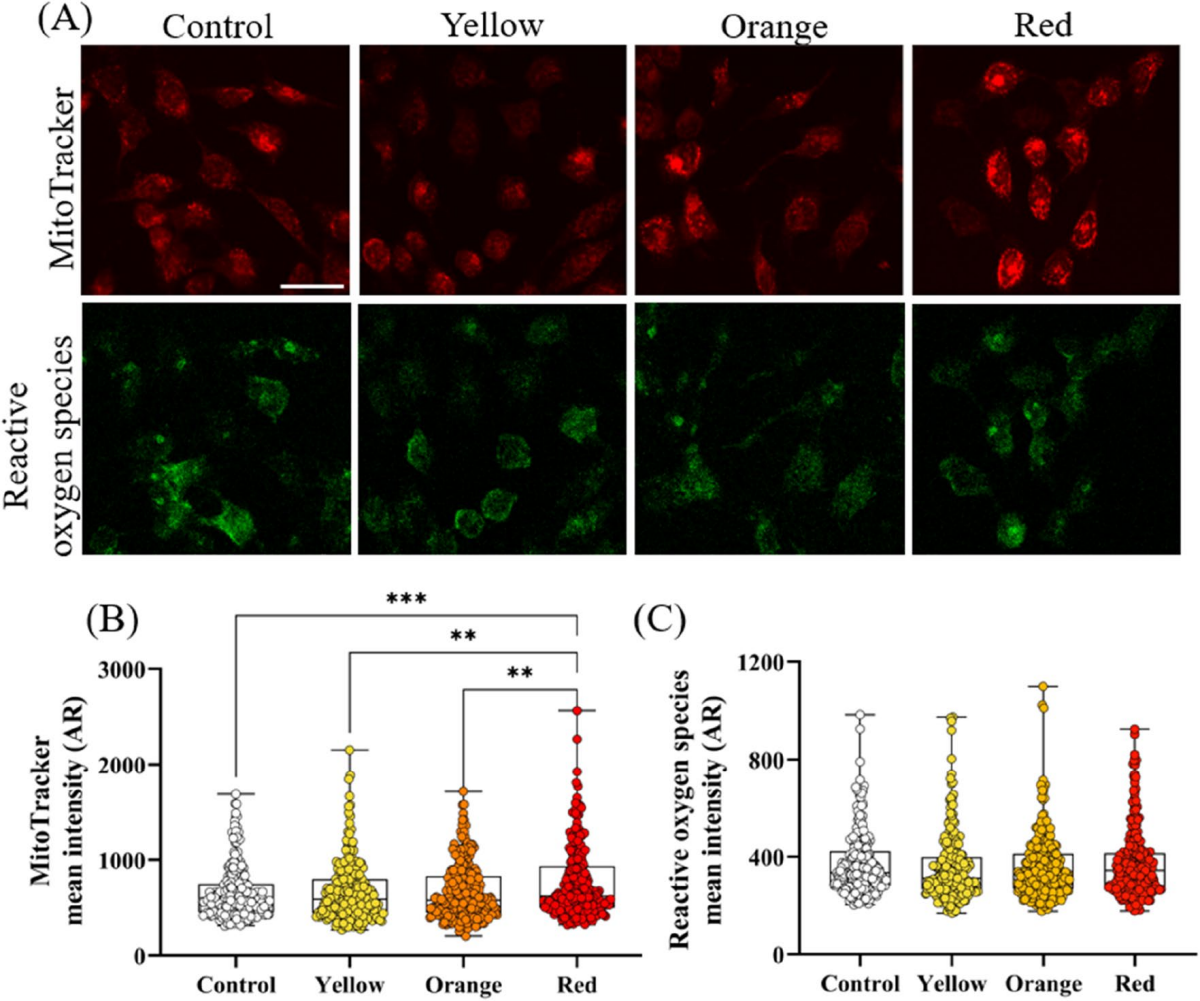
Effect of different wavelengths of visible light radiation on mitochondria membrane potential and oxidative stress in HaCaT cells. (**A**) Immunofluorescent image of MitoTracker and reactive oxidative stress stains (**B**) Quantification of fluorescence intensity of MitoTracker .(**C**) Quantification of fluorescence intensity of oxidative stress. In (**B**,**C**) each dot represents a single cell and at least 240 cells across four independent experiments have been analysed. Boxes show median, first and third quartiles. Scale bar in (**A**) represents 50 μm. Error bars in (**B**,**C**) represent standard error, **P < 0.01, ***P < 0.001, ordinary one-way ANOVA multiple comparisons test.

The intensity of MitoTracker and ROS were measured via immunofluorescent imaging, as shown in Fig. 5A, and quantified as shown in Fig. 5B,C, respectively. The MitoTracker was most intense in cells that had been exposed to the red-light condition, with a fold change of 1.17 ± 0.57 (P < 0.001) compared to the control. There was no statistically significant change in MitoTracker intensity for the orange or yellow light exposures. ROS remained stable for all exposure conditions compared to the control, indicating that visible light did not increase ROS production.

## Discussion

The present proof-of-concept study demonstrates the potential application of the proposed LED-based exposure system to investigate the impact of LED light on skin cells in vitro.

A Study by Sperandio et al. demonstrated increased HaCaT proliferation when exposed to 660 nm laser light with higher output power (100 mW) and fluences (3, 6 or 12 J/cm^2^) then those used in the present study^40^. In a similar study, human keratinocyte cells exposed to 655 nm diode lasers (50 mW @ 1, 3, and 5 J/m^2^) showed that a triple treatment at 1 J/m^2^ provided optimal cell viability, while wound closure was optimal at 3 J/m^2^^16^. While the present study used lower output power, fluence, and only a single exposure, the results indicate the delivered energy was sufficient to impart biostimulatory effects comparable to those described.

While there was no change observed in the ratio of live/dead cells between the control groups and exposed cells, there was a statistically significant increase in cellular BrdU intensity when exposed to visible light indicating an increase in proliferative activity. Interestingly, orange light induced a greater increase in proliferation compared to red but did not significantly increase mitochondrial activation or effect cell viability. This is not unexpected as CCO, a protein in the mitochondrial membrane, is targeted only by the red spectral region (∼ 660 nm) and NIR spectrum (∼ 800 nm)^24^. This finding indicates that the increased proliferation observed in both the yellow and orange light exposure groups may be caused via the stimulation of a different molecular pathway compared to red light. While an increase in cellular ROS is often reported in response to visible light stimulation, no change in ROS was observed at the reported energy densities^41^. This may suggest a possible threshold under which cell proliferation is stimulated without increasing ROS production.

The simplified skin model used for this preliminary study provides a useful tool to evaluate cell proliferation and migration. However, simplified 2D monolayer models may not accurately reflect in vivo processes within the human skin^42^. This includes wound healing, which is a dynamic process where multiple cells coordinate to re-establish epithelial barrier function, with crosstalk between keratinocytes and fibroblast in human skin playing a critical role in coordinating the process^43–45^. However, the observed decrease in scratch size with exposure to visible light is consistent with findings from the literature that suggest visible light can reduce wound healing time of skin^31^. Suggested further work may include using a differentiated epidermis model and the inclusion of a functional dermis to better model the interactions between the epidermis and dermis during PBM.

Unlike lasers, or some other LED-based systems, the individual LEDs can be swapped out enabling different wavelengths to be investigated concurrently^32,33^. However, for effective translation of the device for use by biologists, future work may include developing smartphone connectivity and a user interface to facilitate customized exposure conditions and to automate experiments.

The use and validation of LEDs for PBM paves the way to developing wearable exposure systems for next generation wound healing in home and hospital settings^46^. The efficacy of low intensity light may be further investigated on more complex skin models, and if found capable of improving would healing outcomes, may enable the deployment of low-powered LED based wearables^47,48^. We envisage that the present system will facilitate further studies to elucidate the mechanisms behind PBM by enabling multiple exposure conditions to be reliably repeated using an inexpensive robust system.

## Materials and methods

### Characterisation of exposure system

The power output of each LED was quantified regularly throughout the experiments using a USB Power Meter (PM16-140, Thorlabs) to ensure consistency across the samples. Measurements were taken 13 mm from the tip of the LED to reflect the energy delivered to the cells. The subsequent power and energy densities for a 10-min exposure duration were calculated using the following equations:

$${P}_{d}=\frac{P}{A}$$

$${E}_{d}=\frac{P\times t}{A}$$

where *P*_d_ is power density, *P* is average power, *A* is the area of the bottom of the well (1.54 cm^2^), *E*_d_ is energy density, and *t* is time (600 s).

To monitor any change in temperature that may result from the visible light exposure, a thermal camera (FLIR C2, FLIR) was employed. The camera was centred underneath a 24-well plate with 1 ml of water in each well, with images captured every minute over a period of 60 min.

### Cell culture

The spontaneously immortalized human keratinocyte cell line HaCaT were kindly provided by Associate Professor Terry Piva (RMIT University) and were cultured in RPMI medium supplemented with 10% fetal bovine serum, Penicillin–Streptomycin, and l-glutamine (Gibco Invitrogen). Cells were maintained at 37 °C in a humidified incubator with 5% CO_2_ and for all experiments, cells were passaged every 2–3 days and used up to passage 8 in the conditions described above.

### Visible light exposure

24 h after seeding, media was removed from the cells and replaced with 500 μl of PBS to ensure any observed effects were caused by direct simulation of cells, rather than possible changes to the media from light exposure. Once the PBS was added, the LED array was lowered to 13 mm above the HaCaT cells and turned on for 10 min. Immediately post exposure, the PBS was removed and replaced with 1 ml of complete media.

Key information pertaining to the LED dose and beam parameters can be found in Table 1, with a more detailed description, following recommendations from Jenkins et al. found in S3^49^.

**Table 1:** LED exposure system specifications.

Wavelength	660 nm (red)	610 nm (orange)	585 nm (yellow)
Semiconductor material	GaAlAs	AlInGaP	GaAsP
Exposure duration (s)	600	600	600
Measured average power, P, (μW)	80.5	80.11	9.5
Power density/irradiance, Pd, (μW/cm^2^)	52.27	52.02	6.17
Energy density/fluence, Ed, (mJ/cm^2^)	31.36	31.12	3.7

### Immunocytochemistry and confocal microscopy

#### Cell viability assay

To quantify the ratio of dead to alive cells, 48 h after exposure the quantity of non-viable cells were assessed by Propidium iodide (PI) (Sigma) staining at 1/400 ratio to cell media. Followed by that, cells were washed with PBS, three times to remove excess dye followed by fixing the cells. After that total number of nuclei were stained with DAPI (Sigma, 1/400 dilution).

#### Cell proliferation assay

Measurement of cell proliferation was achieved by using the BrdU Cell Proliferation Chemiluminescent Assay Kit (Cell Signalling Technology) following the supplier's instruction. Cells were seeded at a density of 5 × 10^4^ cells/well and incubated for 24 h prior to exposure. Post exposure, the complete media was replaced with BrdU solution diluted in complete media (1/1000 dilution) and the cells returned to the incubator for 48 h to allow for BrdU incorporation. Subsequently, cells were fixed in 4% PFA for 15 min, washed in PBS and denatured (Cell Signalling Technology). After washing twice in PBS, anti-BrdU antibody (Cell Signalling Technology, 1/100 dilution) was added and incubated for 1 h at room temperature. Cells were then washed and blocked with 5% fetal calf serum for 45 min prior to adding anti-mouse 647 secondary antibody (Life Technologies, 1/400 dilution) and incubating for 1 h at room temperature. Finally, the cells were washed twice in PBS and imaged.

#### Scratch assay

HaCaT were plated in 24-well plates at a density of 5 × 10^4^ cells/well and incubated for 48 h to form a confluent monolayer. Followed by that, horizontal and vertical scratches were made using a 1000 μl pipette tip. Scraped cells were removed by washing the monolayer twice using PBS before replacing the RPMI medium. Immediately after washing, the cells were exposed to the visible light conditions for 10 min inside a laminar flow hood. Then, the plates where imaged using a brightfield microscope and camera (Nikon Eclipse TS100, Nikon) and returned to the incubator. Further images were taken at 24 h, 48 h and 72 h after exposure. Cell migration into the scratch wound was achieved by measuring the cell free area using the area measurement tool in ImageJ (https://imagej.nih.gov/ij/).

#### Mitochondrial staining

To measure mitochondrial membrane potential, MitoTracker Orange (Thermofisher Scientific) dye that stains mitochondria in live cells and accumulates with increases in mitochondrial membrane potential was used. For each experiment, HaCaT were seeded at a density of 5 × 10^4^ cells/well in opaque 24-well plates (VisiPlate, Perkin Elmer) and grown to approximately 70% confluence within 24 h. The complete media was then removed, and the cells washed in serum free media. Mitochondria where stained using MitoTracker Orange at 1/5000 dilution for 1 h, then the cells were washed twice with complete media. The cells were then exposed to the visible light conditions (or sham) for 10 min inside a laminar flow hood followed by 48 h incubation prior to imaging.

#### Measurement of reactive oxygen species

The presence of reactive oxygen species was measured using a Fluorometric Intracellular ROS Kit (Sigma) following the supplier's instruction. 48 h post exposure, cells were washed with PBS twice and incubated for 1 h with ROS detection reagent solution. The supernatant was then removed, and the live cells imaged.

#### Image acquisition and analysis

All image acquisition experiments (except for the scratch assay) were performed with a Nikon A1MP Multiphoton microscope controlled by Nikon Elements software (Nikon). For the immunocytochemistry analysis, images were processed to find the intensity of each stain using NIS Elements software (Nikon). The cell free area for the scratch assay was found using ImageJ.

#### Statistical analysis

Statistical differences were evaluated using either one-way ANOVA multiple comparisons tests (viability, proliferation, mitochondrial membrane potential) or two-way ANOVA Tukey’s multiple comparisons test (scratch) with GraphPad Prism 9.2.0 software (GraphPad, San Diego, USA).

## Supplementary Information

Supplementary Information.

## Data Availability

The datasets used and/or analysed during the current study are available from the corresponding author on reasonable request.
