# Supplementary material for: Investigating the effects of low intensity visible light on human keratinocytes using a customized LED exposure system
Source: Sci Rep. 2022 Nov 7;12:18907. doi: 10.1038/s41598-022-23751-3 (PMC9640685; doi:10.1038/s41598-022-23751-3)
Supplement: Supplementary file 1 — Supplementary Information. [file 41598_2022_23751_MOESM1_ESM.docx]

**Supplementary information**

**Investigating the effects of low intensity visible light on human keratinocytes using a customized LED exposure system**

Emily Sutterby ^1^, Chanly Chheang ^2^, Peter Thurgood ^1^,
Khashayar Khoshmanesh ^1^, Sara Baratchi ^2†^, Elena Pirogova ^1†*^

^1^ School of Engineering, RMIT University, Melbourne, Victoria, Australia

^2^ School of Health and Biomedical Sciences, Bundoora, Victoria, Australia

† These authors contributed equally

* Corresponding author: [elena.pirogova@rmit.edu.au](mailto:elena.pirogova@rmit.edu.au)

**Supplementary information 1:** Approximate cost of the exposure device

S1 – Table showing cost of exposure system

| Component | Supplier | cost |
| --- | --- | --- |
| Microscope holder | [Amazon](https://www.amazon.com.au/Sangmei-Microscope-Magnifier-Adjustable-Universal/dp/B09DCTCGHG/ref=sr_1_291?crid=1OES4LZRGUDJU&keywords=microscope+holder&qid=1641514099&sprefix=microscope+holde%2Caps%2C277&sr=8-291) | 20 |
| Prototyping board | [Jaycar](https://www.jaycar.com.au/ic-experimenters-board-140-x-95mm/p/HP9558) | 8.95 |
| Batteries | [Jaycar](https://www.jaycar.com.au/9v-battery-alkaline-eclipse/p/SB2423?pos=2&queryId=4c853c7d78f596f459013de3bea8643c) | 10 |
| LED driver | [Mouser Electronics](https://au.mouser.com/ProductDetail/Maxim-Integrated/MAX16823EVKIT%2b?qs=g%252BEszo6zu8MlvrQWNue2yw%3D%3D) | 45 |
| LEDs | Mouser Electronics | Approx. 0.30 each |

Total: approx. $90 AUD

**Supplementary information 2:** Results from cell viability assay


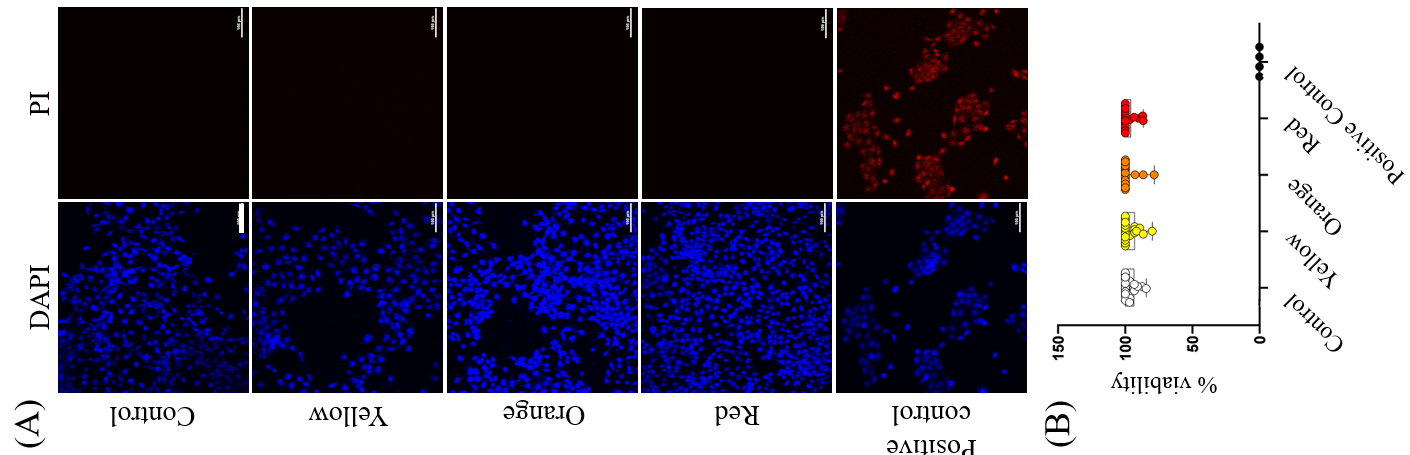


**S2.** Cell viability was measured using a dead/live assay. (A) Immunofluorescent stains on DAPI showing all cells and PI to show dead cells. (B) Quantification of cell viability. Scale bar in A represents 100 μm.

**Supplementary information 3:** Detailed LED dose and beam characteristics.

Table S3.1 - Device information (per well on 24-well plate)

|  | Red LED | Orange LED | Yellow LED |
| --- | --- | --- | --- |
| Manufacturer | Lumex | Lumex | Lumex |
| Model identifier | SSL-LX3044SRD | SSL-LX3044SOD | SSL-LX3044LYD-TR |
| Number of Emitters | 1 | 1 | 1 |
| Emitter Type | GaAlAs | AlInGaP | GaAsP |
| Beam Delivery System | Fixed distance | Fixed distance | Fixed distance |
| Voltage | 1.8V | 1.8V | 1.8V |
| Current delivered | 30mA | 30mA | 30mA |

Table S3.2 - Irradiation parameters

| Parameter [unit] | Red LED | Orange LED | Yellow LED | Measurement method or information source |
| --- | --- | --- | --- | --- |
| Center wavelength [nm] | 660 | 610 | 585 | Manufacturer datasheet |
| Operating mode | Continuous wave | Continuous wave | Continuous wave | - |
| Average radiant power [mW] | 0.80 | 0.80 | 0.09 | USB power meter (PM16-140, Thorlabs) |
| Aperture diameter [mm] | 3 | 3 | 3 | Manufacturer datasheet |
| Viewing angle [deg] | 60 | 60 | 60 | Manufacturer datasheet |
| Beam shape | Circular | Circular | Circular | - |

Table S3.3 - Treatment parameters

| Parameter [unit] | Red LED | Orange LED | Yellow LED | Additional notes |
| --- | --- | --- | --- | --- |
| Beam spot size at target [cm2] | 1.54 | 1.54 | 1.54 | - |
| Irradiance at target [mW/cm2] | 0.52 | 0.52 | 0.06 | Calculated from average radiant power |
| Exposure duration [sec] | 600 | 600 | 600 | - |
| Radiant exposure [J/cm2] | 0.31 | 0.31 | 0.04 | Calculated from average radiant power |
| Radiant energy [J] | 0.48 | 0.48 | 0.06 | Calculated from average radiant power |
| Area irradiated [cm2] | 1.54 | 1.54 | 1.54 | - |
| Application technique | 13mm between LED and cells | 13mm between LED and cells | 13mm between LED and cells | - |
| Number of treatment sessions | 1 | 1 | 1 | - |
| Total radiant energy [J] | 0.48 | 0.48 | 0.06 | Calculated from average radiant power |

**Supplementary information 4:** Transmission and absorption of light through cells, PBS, and plastic

A brief experiment was conducted to reveal absorbance of light through HaCaT cells, PBS, and plastic at the bottom of the 24-well plate.

**Materials and method**

HaCaT cells from passage 15 were cultured in RPMI medium supplemented with 10% fetal bovine serum, Penicillin-Streptomycin, and L-glutamine (Gibco Invitrogen) and maintained at 37°C in a humidified incubator with 5% CO_2_. Cells were seeded in four wells at a density of 5×10^4^ cells/well in opaque 24-well plates (VisiPlate, Perkin Elmer) and incubated for 24 hrs. Then, the plate was removed from the incubator, and the media was replaced with 500 μl of PBS (Cells & PBS & plastic). 500 μl of PBS was then added to four empty wells (PBS & plastic), and four wells were left empty (plastic only). A USB Power Meter (PM16-140, Thorlabs) was then used to record the power output through each well containing PBS, PBS and cells, just the well, or only air. The power meter was placed underneath the well plate, with the LED 13 mm above. The transmittance and absorbance were subsequently calculated using the Beer-Lambert Law:

$$T= \frac{I}{I_{o}}$$

$$A=-log (T)$$

Transmittance, *T*, is the ratio of the transmitted intensity (i.e., the light recorded after passing through the material), *I*, over the incident intensity (i.e., the light intensity prior to hitting the material), $I_{o}$. Absorbance, *A*, is the negative log of transmittance.

Absorbance is a unitless measure of the quantity of light absorbed by a material. It is logarithmically related to transmittance, with and absorbance of 0 indicating that the material does not absorb any light, an absorbance equal to 1 indicating that 90% of the light is absorbed, an absorbance of 2 indicating 99% of light has been absorbed and so on.

**Results**

The average light intensity recorded through the 24-well plate with various materials was recorded and shown in table S4.1. From this, the average light intensity through just PBS and just the cells could be calculated, and the subsequent transmittance percentage and absorbance values are shown in table S4.2 and S4.3, respectively. The results indicate that for all wavelengths, most of the light (>90%) is passed through the solution, meaning that very little light is absorbed by the cells or PBS.

Table S4.1 - Average light intensity as measured by Thorlabs PM16-140

|  | Air only (μW) | Cells & PBS & plastic (μW) | PBS & plastic (μW) | Plastic (μW) |
| --- | --- | --- | --- | --- |
| 660 nm (red) | 80.94 | 73.66 | 75.01 | 76.00 |
| 610 nm (orange) | 81.17 | 72.30 | 74.17 | 75.33 |
| 585 nm (yellow) | 8.33 | 6.77 | 7.20 | 7.81 |

Table S4.2 - Transmittance percentage

|  | Plastic | PBS | Cells |
| --- | --- | --- | --- |
| 660 nm (red) | 93.9 | 98.8 | 98.3 |
| 610 nm (orange) | 92.8 | 98.6 | 97.7 |
| 585 nm (yellow) | 93.7 | 92.8 | 94.7 |

Table S4.3 - Absorbance

|  | Plastic | PBS | Cells |
| --- | --- | --- | --- |
| 660 nm (red) | 0.027 | 0.005 | 0.007 |
| 610 nm (orange) | 0.032 | 0.006 | 0.010 |
| 585 nm (yellow) | 0.028 | 0.033 | 0.023 |
